# A Galaxy-based bioinformatics pipeline for optimised, streamlined microsatellite development from Illumina next-generation sequencing data

**DOI:** 10.1007/s12686-016-0570-7

**Published:** 2016-08-02

**Authors:** Sarah M. Griffiths, Graeme Fox, Peter J. Briggs, Ian J. Donaldson, Simon Hood, Pen Richardson, George W. Leaver, Nathan K. Truelove, Richard F. Preziosi

**Affiliations:** 1 Faculty of Life Sciences, University of Manchester, Manchester, M13 9PT UK; 2 Bioinformatics Core Facility, Faculty of Life Sciences, University of Manchester, Manchester, M13 9PT UK; 3 Research Infrastructure Team, Research Computing, University of Manchester, Manchester, M13 9PL UK; 4 Smithsonian Marine Station, Fort Pierce, FL 34949 USA

**Keywords:** Microsatellite isolation, Pal_finder, PANDAseq, Trimmomatic, Pal_filter, Seq-SSR, SSRs, Galaxy, Next-generation sequencing, Illumina

## Abstract

Microsatellites are useful tools for ecologists and conservationist biologists, but are taxa-specific and traditionally expensive and time-consuming to develop. New methods using next-generation sequencing (NGS) have reduced these problems, but the plethora of software available for processing NGS data may cause confusion and difficulty for researchers new to the field of bioinformatics. We developed a bioinformatics pipeline for microsatellite development from Illumina paired-end sequences, which is packaged in the open-source bioinformatics tool Galaxy. This optimises and streamlines the design of a microsatellite panel and provides a user-friendly graphical user interface. The pipeline utilises existing programs along with our own novel program and wrappers to: quality-filter and trim reads (Trimmomatic); generate sequence quality reports (FastQC); identify potentially-amplifiable microsatellite loci (Pal_finder); design primers (Primer3); assemble pairs of reads to enhance marker amplification success rates (PANDAseq); and filter optimal loci (Pal_filter). The complete pipeline is freely available for use via a pre-configured Galaxy instance, accessible at https://palfinder.ls.manchester.ac.uk.

## Introduction

Microsatellites are popular and effective genetic markers that are utilised in many conservation genetics studies and can inform natural resource management (for example, Maudetr et al. 2002; Jehle and Arntzen 2002; Truelove et al. 2014). Their high rate of polymorphism, codominant mode of inheritance and their utility with even degraded DNA make microsatellites a go-to marker for many studies in ecology and conservation (Sunnucks 2000; Selkoe and Toonen 2006). However, these markers are taxa-specific, meaning primers must often be developed de novo for each new species or genus—traditionally an expensive and time-consuming process.

High-throughput next-generation sequencing (NGS) has decreased the cost-per-base of DNA sequencing significantly, while massively increasing the output (Wetterstrand 2012). Where random enrichment strategies were once used to target microsatellites, new methods to detect short sequence repeats (SSRs) directly from NGS datasets are being developed; the so-called Seq-SSR approach (Goldstein and Schlotterer 1999; Castoe et al. 2012). It is now cost- and time-effective to perform shotgun genome sequencing, computationally identify SSRs in the raw sequencing reads and search their flanking regions for potential primer binding sites (Zalapa et al. 2012). Further cost reductions can be achieved by using Illumina paired-end sequencing, which involves sequencing from both ends of a read (Castoe et al. 2012). This gives greater read lengths than single-end sequencing (up to 2 × 300 base pairs [bp] with the Illumina MiSeq [Illumina 2016]) whilst at a cheaper cost per base than Roche 454 sequencing technology.

The reduced cost, increased number of loci, and more efficient development processes that NGS methods offer mean that microsatellite characterisation is now available to research groups that may have originally been too constrained by cost and time. However, effectively processing the huge amount of data resulting from an NGS run can be challenging for groups without bioinformatics support or previous experience with NGS data. The number of programs available can be daunting, and many can be complicated and time-consuming for novices to master.

**Fig. 1:**
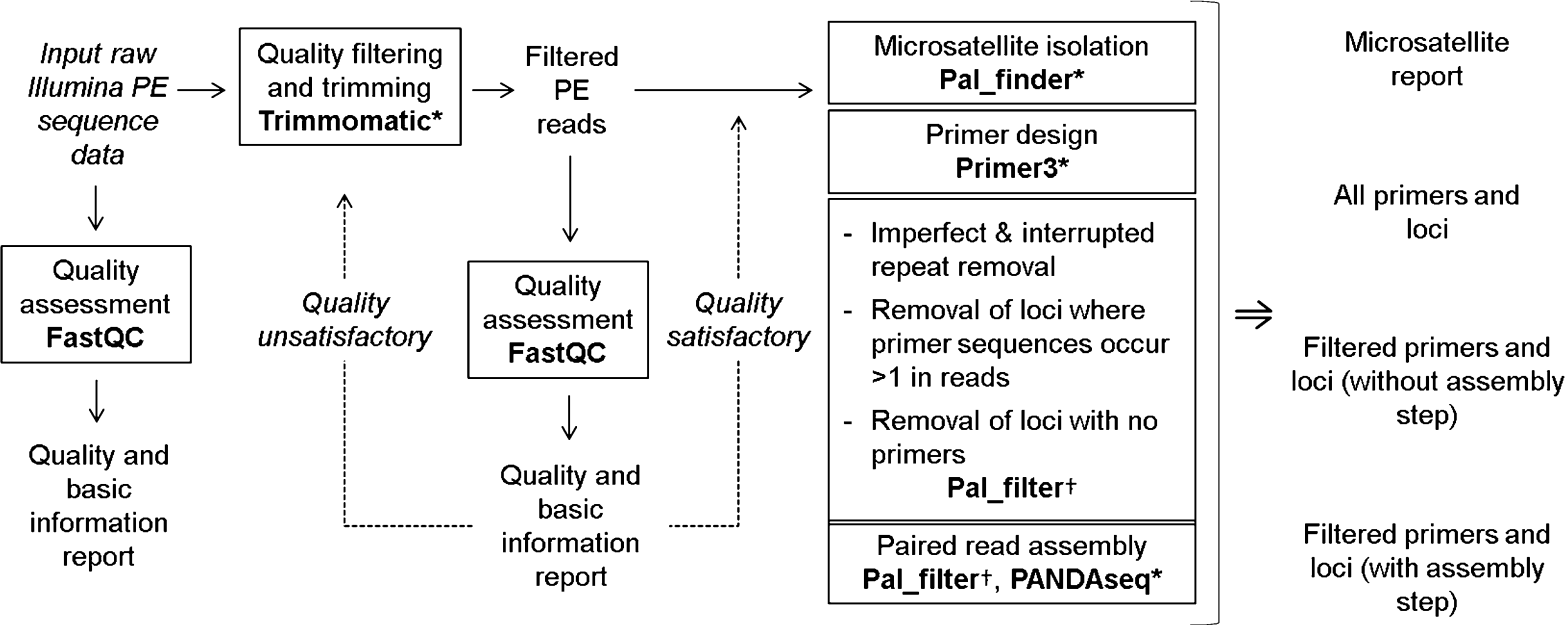
Pipeline processes (*in* *boxes*), the programs used (*in bold*), and pipeline output. * novel wrapper enabling process step to be run in Galaxy;* †* novel program developed by the authors

We have created a complete microsatellite development pipeline for raw Illumina paired-end data that incorporates existing computer programs and a novel filtering script described here (pal_filter). This pipeline has been developed within Galaxy, an open-source, web-based and user-friendly bioinformatics tool for handling large data sets, available on a free public server or to be downloaded as a local installation (Giardine et al. 2005; Blankenberg et al. 2010; Goecks et al. 2010). The use of Galaxy allows the programs within the pipeline be run in a single operational framework, streamlining the process, and providing a graphical user interface (GUI) to increase operational ease and accessibility. Galaxy is well supported, with video tutorials available to support first-time users in use and navigation (see http://galaxyproject.org). Our pipeline provides a complete workflow from receipt of raw sequencing files to production of a list of filtered, optimised microsatellite loci and primers with no further software required for preliminary or post processing (Fig. 1).

## Microsatellite development pipeline processes

### Generating Illumina sequence data

This data-processing pipeline has been developed and optimised for Illumina paired-end sequence data. A single sample should be sequenced for each species intended for microsatellite development. Due to the large volume of data and potential microsatellite primers generated in a single sequencing run, more than one sample can be multiplexed in the same Illumina flow cell lane to allow microsatellite characterisation for multiple species for the same initial sequencing costs (Castoe et al. 2012; also see Table 1). The number of species that can be sequenced in one Illumina flow cell lane whilst still retaining an adequate number of suitable microsatellite primers depends on many factors, including the output capacity of the sequencer, microsatellite-richness of the genomes of the organisms and the types of microsatellite repeats the researchers are interested in (for example, dinucleotide repeats are more common in genomes than longer length repeats). We would advise potential users to consult a sequencing technician before making this choice.

**Table 1:** Case studies of microsatellite development using the described pipeline

Species	P	No. reads (2×)		No. loci with primers [total no. loci]		S_T_ [S_G_] (%)
		Raw	Filtered	Raw reads	Filtered reads	
*Amietia hymenopus* (Phofung river frog)	0.5	6,465,564	3,756,407	25,427 [149,271]* 1,345^†^ 216^‡^	11,350 [60,378]* 1097^†^ 144^‡^	56 [64]
*Raja undulata* (Undulate ray)	0.5	11,019,590	10,174,420	267,431 [130,894]* 3119^†^ 428^‡^	107,470 [31,876]* 342^†^ 148^‡^	73 [80]
*Modiolus modiolus* (Northern horsemussel)	0.125	4,647,211	4,455,417	64,489 [44,408]* 1650^†^ 225^‡^	39,232 [16,814]* 707^†^ 144^‡^	53 [74]

All sequencing was paired end, carried out on the Illumina MiSeq, with sequence lengths of 2 × 250 bp. Trimmomatic settings (SLIDING WINDOW: WINDOW SIZE = 4 bp, QUALITY = 20; LEADING = 3; TRAILING = 3; MINLEN = 50) and primer design conditions (recommended settings for Qiagen Type-it^®^ Microsatellite PCR kit) were constant across all tests. Minimum number of microsatellite repeats to be searched for was eight for all repeat types (2-6mer)

P, proportion of Illumina flow cell lane used; * without pal_filter or assembly; ^†^ with pal_filter (all filtering options selected), without assembly; ^‡^ with pal_filter (all filtering options selected) and assembly; S_T_, total amplification success rate − percentage of loci tested that resulted in amplifiable loci that could be easily scored when fluorescently labeled and analysed using an automated capillary sequencer; S_G_, amplification success rate using agarose gel electrophoresis − percentage of loci tested that resulted in clear bands when visualising PCR products of unlabeled primers on an agarose gel. Primers used in this test were developed from Trimmomatic-fitered reads, with all of the pal_filter and assembly options selected

A number of Illumina platforms are available, which offer users various read length, sequencing output and cost combinations (Illumina 2016). Longer read lengths are advantageous for microsatellite development purposes, as they allow more opportunity for suitable primer binding sites to be found in the microsatellite flanking regions. However, longer reads often suffer from reduced quality at their ends, and therefore they may have to be trimmed to ensure adequate quality (see ‘Quality filtering of data’ section, below). Additionally, longer read lengths allow for primers for larger PCR amplicons to be designed, which can be more prone to large allele dropout (Sefc et al. 2003). Currently, the MiSeq platform allows a maximum read length of 2 × 300 bp (Illumina 2016). However, Castoe et al. (2012) successfully used 2 × 116 bp read lengths generated by the GAIIx platform to develop microsatellite primers. As sequencing technology is constantly evolving, again we would recommend users to consult a sequencing technician to discuss the most appropriate platform and read length to use.

### Quality filtering of data

Data resulting from automated sequencing processes inevitably contains error (especially at the end of reads), which can negatively affect downstream applications. In microsatellite development, miscalled bases in the microsatellite flanking regions could lead to ineffective primer design, non-binding or mis-priming with the target sequence during PCR, and subsequent amplification failure.

We have incorporated Trimmomatic v.0.32 (Bolger et al. 2014) into the pipeline to trim low-quality bases from reads and remove low-quality reads. Specially formulated for paired-end data, Trimmomatic discards both members of a pair if either one does not pass user-specified quality thresholds. This ‘pair-awareness’ results in two files in which the parity of the paired end reads is maintained, essential for the correct functioning of programs downstream. Users can also use Trimmomatic to remove adapter sequences from the reads that have been left over from the sequencing process.

### Read quality and basic information report

FastQC v0.11.4 (Andrews 2014) is used to generate reports containing basic statistics on the reads and various quality assessments. Reports are generated both from the raw and quality-filtered data files, containing useful information such as Phred (quality) scores, GC content, sequence duplication levels, sequence length distribution, and amount and type of adapter content.

### Microsatellite identification and primer design

The files containing surviving pairs from the Trimmomatic process are used for identification of microsatellites and PCR primer design. Sequences containing repeat motifs of up to 6 bp are identified using the program Pal_finder v.0.02.04 (Castoe et al. 2012). The program then examines the flanking regions for suitability as PCR priming sites (identifying ‘PALs’; potentially amplifiable loci), and if suitable, uses Primer3 (Koressaar and Remm 2007; Untergasser et al. 2012) to design primers according to parameters specified by the user (for example, melting temperature and primer length). Two tab delimited files are outputted (readable by Microsoft Excel); one comprising a list of the types of microsatellites found, and another giving a list of all the loci found including the motif, primer sequence, number of occurrences of the primer sequence in the total reads, and the sequence IDs of the forward and reverse reads.

### Microsatellite loci filtering

We incorporated a series of optional filters into the pipeline (implemented via a novel Python script, which we have named Pal_filter) to select the optimal loci from the Pal_finder output text file of microsatellite loci and primers. This gives the user the option to filter out any or all of the following: (1) Loci for which primers could not be designed by Primer3; (2) Loci with imperfect or interrupted motifs (as these do not follow the stepwise mutation model, which many microsatellite population genetics analysis programs assume). If enabled, the loci are also ranked by size of motif (largest first); (3) Loci in which the primer sequences occur more than once in the total reads (to ensure a copy number of one and avoid genes with duplication in the genome). This generates an easy to navigate, tab delimited file and negates the need for manual sorting of potentially thousands of results from the original Pal_finder output. The original file of all PALs and primers is still available (as are all outputs from the pipeline).

### Improving PCR success: paired read assembly

Despite the many benefits of NGS workflows, pairs of primers must still be manually tested in the laboratory to ensure successful amplification. This can represent a considerable cost in both time and resources in the development of a panel of working microsatellite markers. We implemented an additional quality-filtering step with the specific aim of improving the rate of successful PCR and thus reducing these expenses. In brief, the paired-end read assembler PANDAseq (Masella et al. 2012) is used to provide confirmation that both primer sequences occur in the same region of DNA template and increase PCR success (Fox et al. unpublished). This additional quality check is implemented as part of the Pal_filter script. Selecting this option will generate another tab delimited file that again reduces the Pal_finder output to those loci in which the reads could be assembled, as well as incorporating any of the previous filters that have been applied (while still retaining all the other output files).

## Case studies

Table 1 shows the number of microsatellites primers found and subsequent amplification success rates for a variety of configuration options in three species across different taxa (an amphibian, an elasmobranch and a mollusc). Total amplification success rates (S_T_; percentage of primers tested that resulted in loci that were amplifiable and scorable by capillary electrophoresis) ranged from 53 to 73 %, providing proof of principle that the pipeline described here consistently results in successful microsatellite primer development. Table 1 also shows the percentage of primers tested that produced PCR products that could be visualised using agarose gel electrophoresis (S_G_); it should be noted that this is consistently higher than the total amplification success rate. We have reported this to highlight that initial testing of primers on agarose gels may not reflect the actual number of usable loci that will be available when using capillary electrophoresis to measure allele sizes. This can be due to a number of reasons, including high levels of ‘stutter’ for a locus making the true allele difficult to distinguish, or non-specific binding resulting in multiple peaks on a sequencer trace.

The case studies also highlight the potential economy of this method. *Modiolus modiolus* was sequenced in an Illumina flowcell lane with seven other species for microsatellite development purposes, and 144 loci with primers were available after the most stringent filtering and assembly options were used. If the total amplification success rate for this species (53 %) is assumed to apply for all these loci, this would still mean that around 76 loci would be usable in a conservation genetics study. Currently, this far exceeds the number of microsatellites normally used for these purposes. This shows that pooling multiple samples in one lane of an Illumina flowcell can reduce the cost-per-species of microsatellite development considerably whilst still retaining an ample amount of high-quality loci.

Filtering the reads using Trimmomatic removed between 4.1 and 41.9 % (*Raja undulata* and *Amietia hymenopus* respectively) of the raw reads. The settings used (see Table 1) ensured that the remaining reads had an average Phred score of 20 across every four bases, meaning a base call accuracy of 99 %. It is prudent to remove low quality reads and bases in order to reduce the likelihood of designing primers based on miscalled bases, as this may result in PCR amplification failure. This effect could be substantial when a high proportion of reads are low quality (as in *Amietia hymenopus*).

## Summary

This bioinformatics pipeline is a robust method for designing effective microsatellite primers, and its incorporation into Galaxy provides a user-friendly framework in which to operate the pipeline. Our lab group has successfully used this method to develop microsatellite markers in a number of species, including vertebrates (Bertolotti et al. 2015), invertebrates and plants (data unpublished, also see Table 1).

As microsatellite development becomes more accessible to researchers, it is important to consider both the positive and negative aspects of microsatellites as molecular markers before embarking on development projects. A number of articles discuss these potential issues (for example, Selkoe and Toonen 2006; Väli et al. 2008; Guichoux et al. 2011; Putman and Carbone 2014) and should be reviewed by any potential microsatellite users. Users of the pipeline described here are also encouraged to consult the articles cited for each of the programs utilised, as well as the user manual for the pipeline (see https://palfinder.ls.manchester.ac.uk/manual), which goes into detail on user-specified settings and use of the programs in Galaxy. We envision that this will be a useful tool for both academic and non-academic groups involved in conservation genetics research due to its comprehensiveness, effectiveness and ease of use.

## Accessing the pipeline

There are three options available for potential users: (1) A public Galaxy instance (called Galaxy Palfinder Service) implementing the pipeline with complete functionality as described here is available online for research use at https://palfinder.ls.manchester.ac.uk. A manual including detailed instructions for use is available at https://palfinder.ls.manchester.ac.uk/manual; (2) Advanced users with access to their own local Galaxy server may download the Trimmomatic and Pal_finder (including Pal_filter) wrappers from https://toolshed.g2.bx.psu.edu/view/pjbriggs/, and the FastQC wrapper from https://toolshed.g2.bx.psu.edu/view/devteam/fastqc/; (3) Finally, all programs can be run outside the Galaxy environment at the command line (Unix) (for detailed instructions, see user manual).

## References

[CR1] Andrews S (2014) FastQC: a quality control tool for high throughput sequence data [Online]. http://www.bioinformatics.babraham.ac.uk/projects/fastqc/. Accessed 7 April 2016

[CR2] Bertolotti AC, Griffiths SM, Truelove NK, Box SJ, Preziosi RF, Salinas de Leon P (2015). Isolation and characterization of 10 polymorphic microsatellite loci for the endangered Galapagos-endemic whitespotted sandbass (*Paralabrax albomaculatus*). PeerJ.

[CR3] Blankenberg D, Von Kuster G, Coraor N, Ananda G, Lazarus R, Mangan M, Nekrtenko A, Taylor J (2010). Galaxy: a web-based genome analysis tool for experimentalists. Curr Protoc Mol Biol.

[CR4] Bolger AM, Lohse M, Usadel B (2014). Trimmomatic: a flexible trimmer for Illumina sequence data. Bioinformatics.

[CR5] Castoe TA, Poole AW, de Koning J, Jones KL, Tomback DF, Oyler-McCance SJ, Fike JA, Lance SL, Streicher JW, Smith EN, Pollock dd (2012). Rapid microsatellite identification from Illumina paired-end genomic sequencing in two birds and a snake. PLoS ONE.

[CR6] Giardine B, Riemer C, Hardison RC, Burhans R, Elnitski L, Shah P, Zhang Y, Blankenberg D, Albert I, Taylor J, Miller W, Kent WJ, Nekrutenko A (2005). Galaxy: a platform for interactive large-scale genome analysis. Genome Res.

[CR7] Goecks J, Nekrutenko A, Taylor J, The Galaxy Team (2010). Galaxy: a comprehensive approach for supporting accessible, reproducible and transparent computational research in the life sciences. Genome Biol.

[CR8] Goldstein DB, Schlotterer C (1999). Microsatellites: evolution and applications.

[CR9] Guichoux E, Lagache L, Wagner S, Chaumeil P, Léger P, Lepais O, Lepoittevin C, Malausa T, Revardel E, Salin F, Petit RJ (2011). Current trends in microsatellite genotyping. Mol Ecol Resour.

[CR10] Illumina (2016) Illumina next-generation sequencing platforms. http://www.illumina.com/systems/sequencing-platform-comparison.html. Accessed 7 April 16

[CR11] Jehle R, Arntzen J (2002). Review: microsatellite markers in amphibian conservation genetics. Herpetol J.

[CR12] Koressaar T, Remm M (2007). Enhancements and modifications of primer design program Primer3. Bioinformatics.

[CR13] Masella AP, Bartram AK, Truszkowski JM, Brown DG, Neufeld JD (2012). PANDAseq: paired-end assembler for Illumina sequences. BMC Bioinf.

[CR14] Maudetr C, Miller C, Bassano B, Breitenmoser-Würsten C, Gauthier D, Obexer-Ruff G, Michallet J, Taberlet P, Luikart G (2002). Microsatellite DNA and recent statistical methods in wildlife conservation management: applications in Alpine ibex [*Capra ibex*(*ibex*)]. Mol Ecol.

[CR15] Putman AI, Carbone I (2014). Challenges in analysis and interpretation of microsatellite data for population genetic studies. Ecol Evol.

[CR16] Sefc KM, Payne RB, Sorenson MD (2003). Microsatellite amplification from museum feather samples: effects of fragment size and template concentration on genotyping errors. Auk.

[CR17] Selkoe KA, Toonen RJ (2006). Microsatellites for ecologists: a practical guide to using and evaluating microsatellite markers. Ecol Lett.

[CR18] Sunnucks P (2000). Efficient genetic markers for population biology. Trends Ecol Evol.

[CR19] Truelove NK, Griffiths S, Ley-Cooper K, Azueta J, Majil I, Box SJ, Behringer DC, Butler MJ, Preziosi RF (2014). Genetic evidence from the spiny lobster fishery supports international cooperation among Central American marine protected areas. Conserv Genet.

[CR20] Untergasser A, Cutcutache I, Koressaar T, Ye J, Faircloth BC, Remm M, Rozen SG (2012). Primer3—new capabilities and interfaces. Nucleic Acids Res.

[CR21] Väli Ü, Einarsson A, Waits L, Ellegren H (2008). To what extent do microsatellite markers reflect genome-wide genetic diversity in natural populations?. Mol Ecol.

[CR22] Wetterstrand KA (2012) DNA sequencing costs: data from the NHGRI large scale genome sequencing program. http://www.genome.gov/SequencingCosts/. Accessed 13 Feb 2016

[CR23] Zalapa JE, Cuevas H, Zhu H, Steffan S, Senalik D, Zeldin E, McCown B, Harbut R, Simon P (2012). Using next-generation sequencing approaches to isolate simple sequence repeat (SSR) loci in the plant sciences. Am J Bot.

